# Loop-mediated isothermal amplification (LAMP) assays for detection of the New Guinea fruit fly *Bactrocera* *trivialis* (Drew) (Diptera: Tephritidae)

**DOI:** 10.1038/s41598-022-16901-0

**Published:** 2022-07-23

**Authors:** Melissa L. Starkie, Elizabeth V. Fowler, Xiaocheng Zhu, Arati Agarwal, Lea Rako, Isarena C. Schneider, Mark K. Schutze, Jane E. Royer, David Gopurenko, Peter Gillespie, Mark J. Blacket

**Affiliations:** 1 Department of Agriculture and Fisheries, Biosecurity Queensland, Brisbane, QLD Australia; 2 New South Wales Department of Primary Industries, Orange, NSW Australia; 3 Department of Jobs, Precincts and Regions, Agriculture Victoria, Bundoora, VIC Australia; 4 Department of Agriculture, Water and the Environment, Cairns, QLD Australia

**Keywords:** Biological techniques, Molecular biology

## Abstract

The cue-lure-responding New Guinea fruit fly, *Bactrocera* *trivialis*, poses a biosecurity risk to neighbouring countries, e.g., Australia. In trapping programs, lure caught flies are usually morphologically discriminated from non-target species; however, DNA barcoding can be used to confirm similar species where morphology is inconclusive, e.g., *Bactrocera* *breviaculeus* and *B.* *rufofuscula*. This can take days—and a laboratory—to resolve. A quicker, simpler, molecular diagnostic assay would facilitate a more rapid detection and potential incursion response. We developed LAMP assays targeting cytochrome *c* oxidase subunit I (COI) and Eukaryotic Translation Initiation Factor 3 Subunit L (EIF3L); both assays detected *B.* *trivialis* within 25 min. The BtrivCOI and BtrivEIF3L assay anneal derivatives were 82.7 ± 0.8 °C and 83.3 ± 1.3 °C, respectively, detecting down to 1 × 10^1^ copies/µL and 1 × 10^3^ copies/µL, respectively. Each assay amplified some non-targets from our test panel; however notably, BtrivCOI eliminated all morphologically similar non-targets, and combined, the assays eliminated all non-targets. Double-stranded DNA gBlocks were developed as positive controls; anneal derivatives for the COI and EIF3L gBlocks were 84.1 ± 0.7 °C and 85.8 ± 0.2 °C, respectively. We recommend the BtrivCOI assay for confirmation of suspect cue-lure-trapped *B.* *trivialis*, with BtrivEIF3L used for secondary confirmation when required.

## Introduction

Tephritid fruit flies (Diptera: Tephritidae) are a highly diverse group of insects that include some of the most significant pests of global horticulture and food security^1^. The subfamily Dacinae contains over 900 described species^2^, with its centre of diversity in the Asia/Pacific/Oceania region^3^. It includes some of the world’s most destructive fruit pests, including *Bactrocera* *dorsalis* (Hendel) (oriental fruit fly) and *Zeugodacus* *cucurbitae* (Coquillett) (melon fly)^4^; but there are many other economically important species that are on National Priority Plant Pest lists^5^.

One destructive dacine pest, the New Guinea fruit fly, *Bactrocera* *trivialis* (Drew) is among these economically significant pests^6^ with records from 17 host plants across ten families, including commercial hosts such as starfruit, orange, guava, mango and chilli^7,8^. *Bactrocera* *trivialis* is native to the island of New Guinea^9^ and is detected and eradicated from the Torres Strait Islands, to the immediate north of Queensland (Australia) every year^10^. As these islands are geographically situated between the Australian mainland and Papua New Guinea, such proximity represents an ongoing threat to Australia’s biosecurity^4^.

Australia’s first line of defence against *B.* *trivialis* is monitoring by using a network of traps in the Torres Strait Islands baited with male-specific cue-lure^11^. These lure traps also attract many non-target species^2^, and during the summer trapping season, individual traps may capture over 30,000 flies during a two-week trapping period (unpubl. data, Northern Australian Quarantine Survey). Note that of the lures used in monitoring, *Bactrocera* species do not typically respond to more than one type of lure^12^, therefore cue-lure trap composition is usually predictable. Identification of *B.* *trivialis* among cue-lure trap contents can be difficult due to the presence of morphologically similar non-targets such as *Bactrocera* *breviaculeus* (Hardy) and *Bactrocera* *rufofuscula* (Drew & Hancock). This can pose challenges to rapid and accurate diagnostics.

Genetically, evidence suggests that *B.* *trivialis* is most closely related to the non-commercially important species *Bactrocera* *barringtoniae* (Tryon) and *Bactrocera* *parabarringtoniae* Drew & Hancock^13^. These species do not respond to cue-lure, but instead respond to other male lures, methyl-isoeugenol and methyl eugenol respectively^9,14,15^. Therefore, whilst genetically similar, these species are not encountered in the same surveillance trap as *B.* *trivialis,* as they respond to different lures, and only one lure is ever used per trap.

*Bactrocera* *trivialis* is currently identified from trap catches under a dissecting microscope. Conventional polymerase chain reaction (PCR) and COI barcode sequencing is used to confirm suspect *B.* *trivialis* where the identity is not clear from morphological examination^16^. Both techniques require specialist training and must be undertaken in a laboratory. Identification of *B.* *trivialis* via restriction fragment length polymorphism (RFLP) is possible, but there are no species-specific enzymes that are diagnostic for *B.* *trivialis* and a combination of different enzymes are required to reach a determination^16^.

A range of diagnostic mitochondrial and nuclear loci have recently been used for identifying dacines^17,18^; often in conjunction with traditional cytochrome *c* oxidase subunit I (COI) DNA barcode sequencing to resolve cryptic species^16^. The nuclear eukaryotic translation initiation factor 3 subunit I (EIF3L) region has been particularly effective in diagnosis of difficult species groups^16^. However, none of the alternative loci developed have been used to-date in rapid diagnostic assays (i.e., a result obtained per specimen in under an hour) for identification of any fruit fly species, and there are no rapid or in-field tools available for diagnosis of *B.* *trivialis*. There is, therefore, a need for a simple, rapid, and accurate diagnostic tool that will provide confident identifications in lieu of time-consuming and highly specialised laboratory processes.

Loop-mediated Isothermal Amplification (LAMP) is a tool that is highly specific and suitable for rapid laboratory and in-field diagnostics^19^. A LAMP reaction generally utilises three primer pairs that target eight regions of a chosen DNA fragment^20^. During LAMP reactions, these primers produce stem-loop structures that enable a faster reaction time when compared to conventional PCR^19,20^. Additionally, isothermal conditions required for LAMP reactions can be achieved using a portable, battery operated heating device; thus enabling in-field diagnosis^21^. Consequently, LAMP assays have recently been successfully designed and implemented for the diagnosis of several insect pests^22–25^, including the Queensland fruit fly (*Bactrocera* *tryoni* (Froggatt)) an Australian dacine pest species of economic concern^26^.

Previous studies have designed gBlocks™ Gene Fragments (IDT, USA), synthetic fragments for use as positive controls in LAMP assays^23,24,27^. Implementing a gBlock can provide many benefits depending on the application, including: removing the need for cloning^28^, providing an indication of reaction efficiency^27^, having a readily available, stable positive at a known concentration, and if designed to anneal at a different temperature, can be easily differentiated from sample DNA^25^. In circumstances such as this where *B.* *trivialis* DNA stocks are difficult to obtain for use as positive controls, gBlock gene fragments offer a stable and reliable alternative.

Given the alternative diagnostic loci for the dacines are at our disposal^18^, the extensive sequence data already publicly available^29^, and reported successes using LAMP assays for identifying pest dacines in the past^21,26^; the aim of this research was to: (i) produce species-specific LAMP assays for rapid diagnosis of adult *B.* *trivialis* in the laboratory or field; (ii) design and test the suitability of a synthetic gBlock fragment as a positive control^30^; and (iii) test against a panel of non-target species including morphologically similar, genetically similar, and commonly encountered fruit flies.

## Results

### LAMP primers, assay performance and panel testing

**Figure 1:**
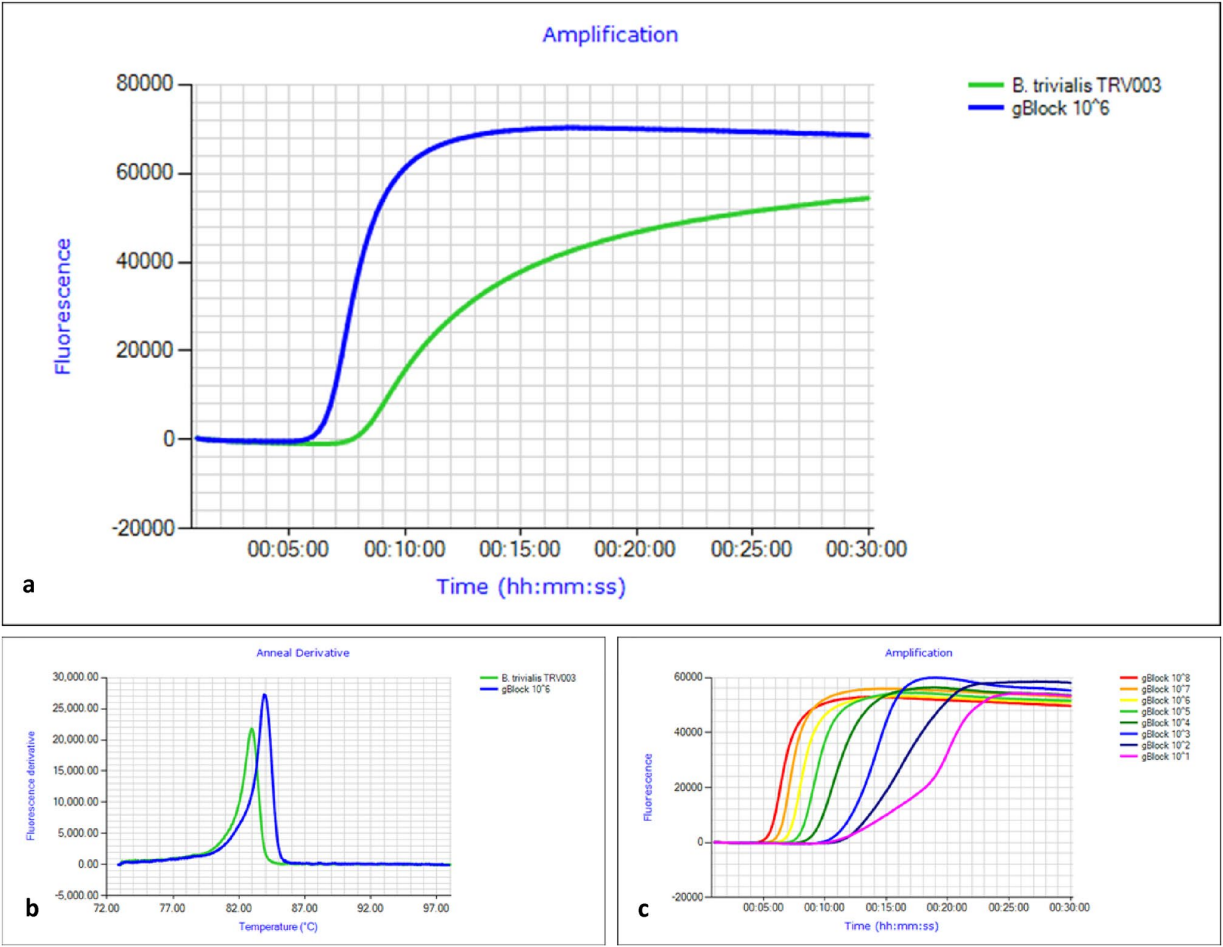
(**a**) Comparison of amplification of gBlock positive control and *Bactrocera* *trivialis* TRV003 DNA in the BtrivCOI LAMP assay. (**b**) Anneal derivative of gBlock positive control and *Bactrocera* *trivialis* TRV003 DNA in the BtrivCOI LAMP assay; in thisLAMP run the gBlock positive anneals at ~ 84 °C, while *B.* *trivialis* DNA anneals at ~ 83 °C. The gBlock positive 1 × 10^6^ copies/µL is the appropriate concentration for use as a control. (**c**) BtrivCOI assay gBlock standard curve run with serial dilutions from 1 × 10^8^ copies/µL to 1 × 10^1^ copies/µL.

**Figure 2:**
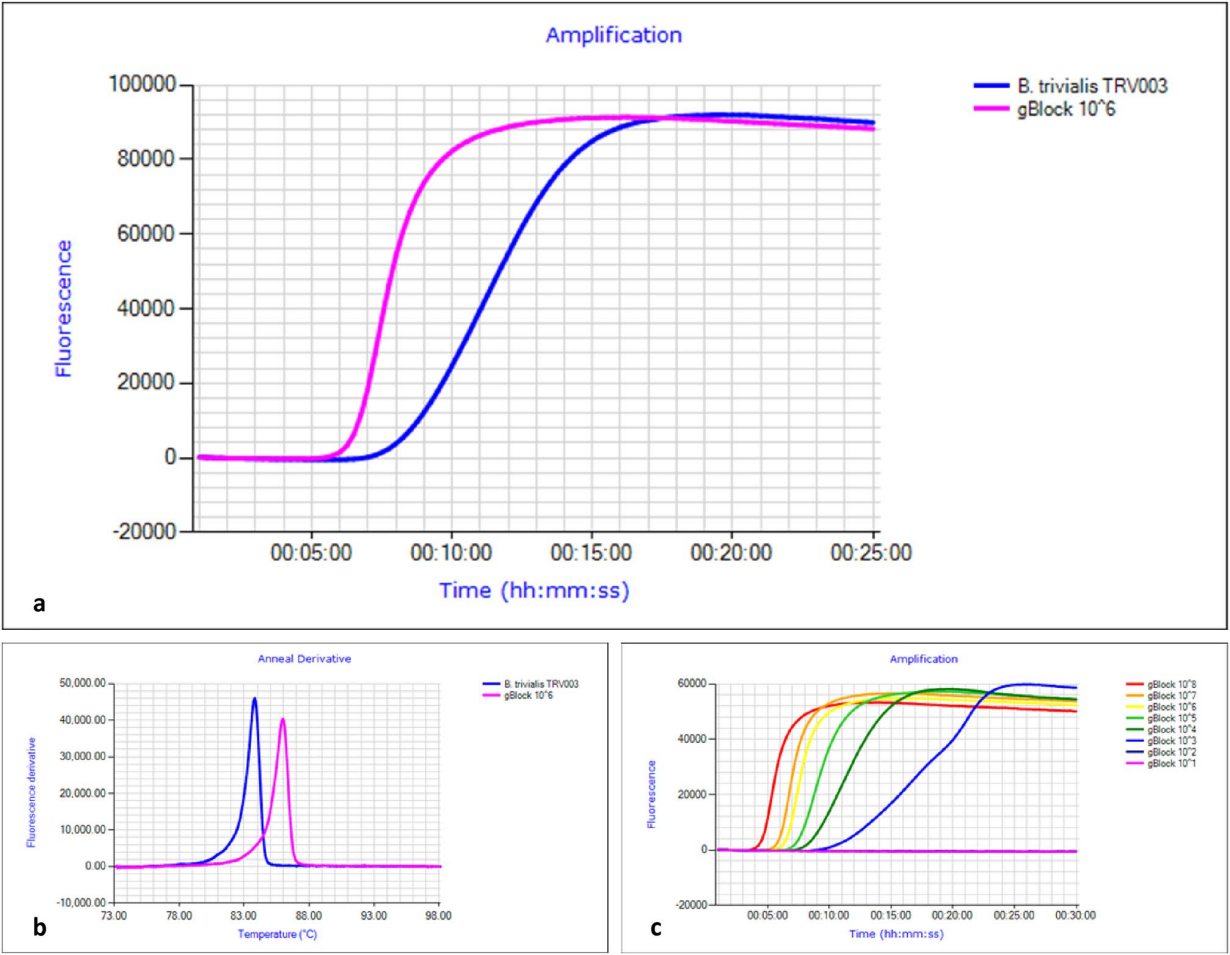
(**a**) Comparison of amplification of gBlock positive control and *Bactrocera* *trivialis* TRV003 DNA in the BtrivEIF3L LAMP assay. (**b**) Anneal derivative of gBlock positive control and *Bactrocera* *trivialis* TRV003 DNA in the BtrivEIF3L LAMP assay; in this LAMP run the gBlock positive anneals at ~ 86 °C, while *B.* *trivialis* DNA anneals at ~ 84 °C. The gBlock 1 × 10^6^ copies/µL is the appropriate concentration for use as a control. (**c**) BtrivEIF3L assay gBlock standard curve run with serial dilutions from 1 × 10^8^ copies/µL to 1 × 10^1^ copies/µL.

We designed two complementary LAMP assays for diagnosis of *B.* *trivialis* adults. Here we refer to the two assays as the BtrivCOI assay, and the BtrivEIF3L assay. For both assays, the F3/B3:FIP/BIP:Floop/Bloop primer pairs (Table 1) were optimal in a ratio of 1:6:3, at final concentrations of 0.4 µM, 2.4 µM and 1.2 µM respectively. The BtrivCOI assay was capable of amplifying *B.* *trivialis* within the 25 min run time, with an anneal derivative of 82.6 ± 0.7 °C (Fig. 1). Of all the non-target species only *B.* *parabarringtoniae* and *B.* *barringtoniae* amplified (< 20 min), while we observed early to late amplification (> 20 min) of *B.* *manskii* (between 17 and 24 min) (Table 2) in the BtrivCOI assay. The BtrivEIF3L assay amplified *B.* *trivialis* samples within the 25 min run time (Fig. 1) with an anneal derivative of 83.3 ± 1.3 °C which was consistent across validating laboratories; and provides a consistent benchmark to compare to the gBlock (Fig. 2). This assay was not as specific as the BtrivCOI assay, but notably, *B.* *parabarringtoniae* and *B.* *barringtoniae* were not amplified in this assay. Other species in the test panel that produced early amplification were *B.* *breviaculeus* and *B.* *rufofuscula,* *B.* sp. near *trivialis* (three morphologically similar species), *B.* *aquilonis,* *B.* *cacuminata,* *B.* *kraussi,* *B.* *musae,* *B.* *opiliae,* and late amplification (> 20 min) of *B.* *peninsularis,* *B.* *tryoni*, *B.* *neohumeralis,* *B.* *bancroftii* and *B.* *dorsalis* (Table 2). Most species with > 20 min amplification were not consistent across all individuals tested for that species.

**Table 1:** Sequences and Tm (°C) of primers designed in this study for BtrivCOI and BtrivEIF3L LAMP assays developed for detection of *Bactrocera* *trivialis.*

Assay	Primer name	Primer sequence 5ʹ-3ʹ	Tm (°C)
BtrivCOI assay (260 bp target)	BtrivCOI_F3	GGAAAACGGGGCTGGTACAGGC	62.9
	BtrivCOI_B3	GCTCCTGCTAAAACTGGTAGAGAT	56.3
	BtrivCOI_FIP	GAGATACCRGCTAAGTGGAGTGAACCCTATCATCTGTTATCGCA	65.8
	BtrivCOI_BIP	YTCAATTTTAGGAGCAGTAAATTTCATTGGCTGTTAATACAACTGCTCAG	63.7
	BtrivCOI_FL	AAAATAGCTAGATCAACTGAAGCT	52.2
	BtrivCOI_BL	ACAACAGTAATTAATATACGATCCACA	52.4
BtrivEIF3L assay (176 bp target)	BtrivEIF_F3	TTATCARGCCATTAAAGTACTGG	51.5
	BtrivEIF_B3	GTGCGTTGAATGTACAAGA	50.5
	BtrivEIF_FIP	AAGTTGAAATTGCAGGCAAACCAATAGAAATCCACAAGAA	62.9
	BtrivEIF_BIP	TGTTGGATTTGCATACATGATGATGGGATTTCAGAGAAAGTGCGA	65.6
	BtrivEIF_FL	GGTATATGAGAATACTGCGAC	50.1
	BtrivEIF_BL	CCGTTATGCCGATGC	50.7

The F2 and B2 primer regions of FIP and BIP are underlined.

**Table 2:**
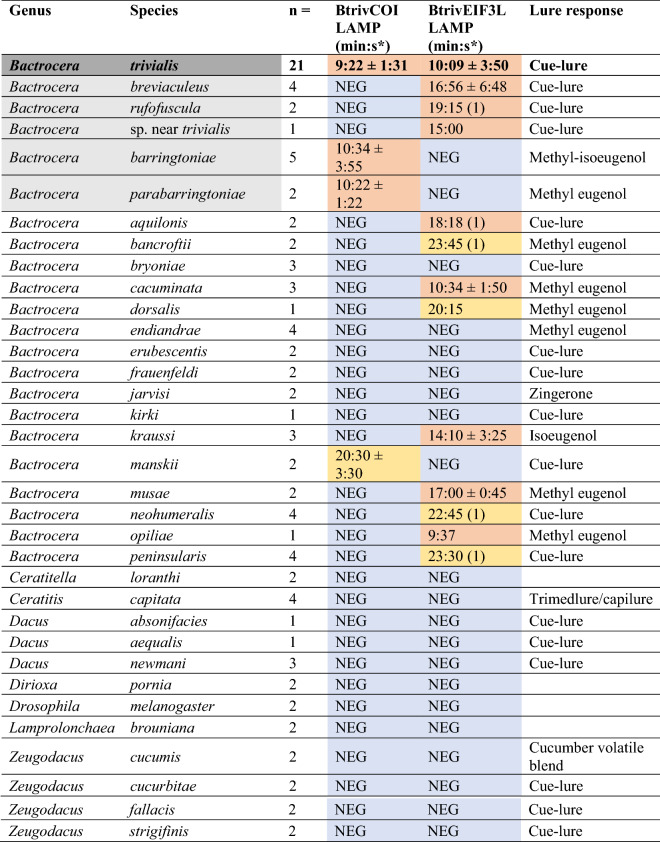
Results from the panel testing for both BtrivCOI and BtrivEIF3L LAMP assays.

Blue = no observed amplification, orange = amplification < 20 min (early), and yellow > 20 min (late); brackets indicate number of samples used to calculate average amplification time, where positive amplification of a species was variable. The target species, and samples morphologically or genetically similar are highlighted in grey; all other species are listed alphabetically. Lure response of each panel species (if any) is also presented^4,15,32^. NB: see supplementary table for individual specimen results.

*Average +/− standard deviation.

### Synthetic gBlock standards and serial dilutions

For the BtrivCOI assay, we found the assay could detect down to 1 × 10^1^ copies/µL with an anneal derivative of 84.1 ± 0.7 °C compared to *B.* *trivialis* DNA, which annealed at 82.6 ± 0.7 °C (Fig. 1). The BtrivEIF3L gBlock detected down to 1 × 10^3^ copies/µL, with an anneal derivative of 85.8 ± 0.2 °C compared to *B.* *trivialis* DNA, which annealed at 83.3 ± 1.3 °C (Fig. 2). In our cross-laboratory validations, we did not observe large deviations in anneal derivatives and sensitivity across laboratories and GENIE III machines. We found that the most appropriate concentration for use as a standard in both assays was the 1 × 10^6^ copies/µL dilution.

BtrivCOI gBlock sequence:

5ʹgggGGAAAACGGGGCTGGTACAGGCgggCCCTATCATCTGTTATCGCAgggAGCTTCAGTTGATCTAGCTATTTTgggTTCACTCCACTTAGCCGGTATCTCgggCTCAATTTTAGGAGCAGTAAATTTCATTgggACAACAGTAATTAATATACGATCCACAgggCTGAGCAGTTGTATTAACAGCCCgggATCTCTACCAGTTTTAGCAGGAGCggg3ʹ.

BtrivEIF3L gBlock sequence:

5ʹgggTTATCAAGCCATTAAAGTACTGGgggAACCAATAGAAATCCACAAGAAgggGTCGCAGTATTCTCATATACCgggTGCCTGCCAAATTTCAACTTgggTGTTGGATTTGCATACATGATGATGgggCCGTTATGCCGATGCgggTCGCACTTTCTCTGAAATCCgggTCTTGTACATTCAACGCACggg3ʹ.

## Discussion

We developed two complementary LAMP assays for accurate and rapid diagnosis of *B.* *trivialis* in the laboratory or field. The BtrivCOI assay is capable of distinguishing *B.* *trivialis* from other morphologically similar adult fruit flies, *B.* *breviaculeus* and *B.* *rufofuscula,* which are attracted to cue-lure. However, when run against our panel of genetically similar, and commonly trapped species, the BtrivCOI assay also amplified *B.* *barringtoniae,* *B.* *parabarringtoniae* and *B.* *manskii.* To address this, we developed a second assay based on the EIF3L locus that amplified some non-target species yet did distinguish between *B.* *trivialis* and the BtrivCOI assay-positive *B.* *barringtoniae,* *B.* *parabarringtoniae* and *B.* *manskii.* The BtrivEIF3L assay offers a reliable secondary test for use on morphologically damaged adult specimens.

Non-specific amplification in the BtrivCOI assay is not of high concern as we expect this assay will predominantly be used to aid in identification of adults caught in cue-lure traps. Since *B.* *parabarringtoniae* is a methyl eugenol responsive species, and *B.* *barringtoniae* responds to methyl- isoeugenol, we do not expect these flies to be present in cue-lure traps. In the case of *B.* *manskii,* it possesses distinctive wing patterning that is absent in *B.* *trivialis* and so would not be used after initial morphological identification for follow up analysis, and this, along with the BtrivEIF3L assay, provides secondary confirmation. Additionally, the two assays can be run optimally under the same reaction conditions and simultaneously, to offer a confident result.

We designed two gBlock standards for use as positive controls in our LAMP assays. We recommend the use of the gBlock standards when implementing the assays for three main reasons: (i) *B.* *trivialis* is an exotic species to Australia, and DNA is often difficult to obtain in large quantities for use as positive controls; (ii) gBlocks are stable, and can be an indicator of primer or mastermix degeneration (particularly important if reagents are freeze-thawed multiple times); and (iii) gBlocks have been designed to have a different Tm from real *B.* *trivialis* DNA so that suspected reaction contaminations by gBlock controls (especially when running the assays in non-sterile field environments) can be easily recognised.

As we did not have access to *B.* *trivialis* larval samples for testing in the assays, the performance of these assays for identification of immatures was not tested. It is expected that immature specimens will be readily detected by both of our assays as they can detect very low copy numbers (1 × 10^1^ for BtrivCOI and 1 × 10^3^ for BtrivEIF3L). That said, it is unlikely that *B.* *trivialis* LAMP assays will be required for immature identification given the differences in host plants used across species. Nevertheless, the performance of similar fruit fly assays which have tested both adult and immature samples have demonstrated LAMP amplification success^26^.

In conclusion, we have developed two assays that in combination, are capable of rapidly identifying adult *B.* *trivialis*. In the absence of any previously developed LAMP or real-time (qPCR) assays, these assays are the first rapid assays developed for this species. Our assays significantly improve on current DNA barcoding methods and incorporate alternative fruit fly diagnostic loci for the first time. Rapid diagnosis of *B.* *trivialis* can now be conducted in under an hour, rather than the typical timeframes required for traditional PCR and sequencing. This also adds to the growing number of species-specific LAMP assays available for fruit flies^21,26^. A fast diagnosis will result in an early management response; ultimately improving biosecurity response capability.

## Materials and methods

### Specimens examined

**Figure 3:**
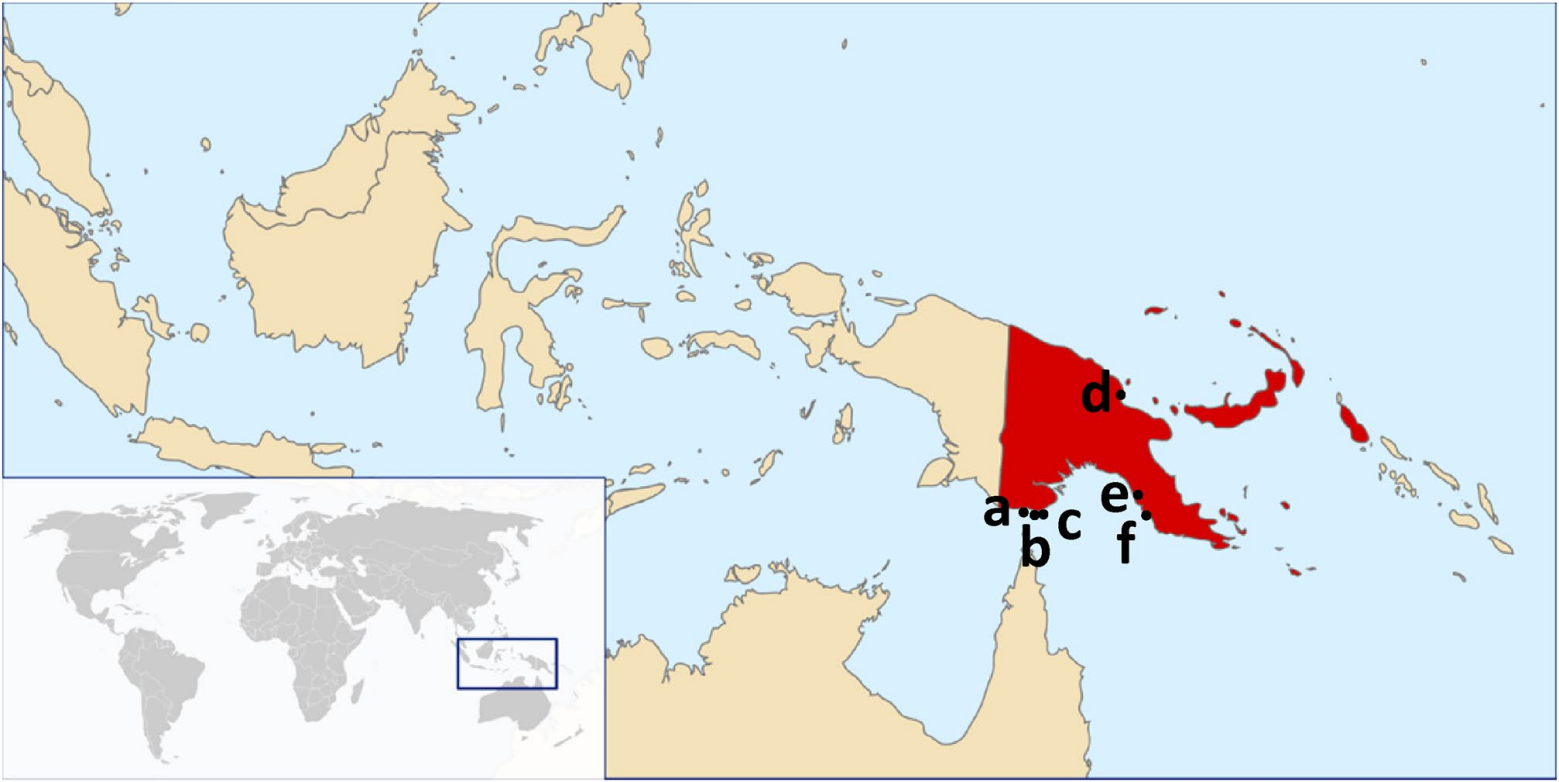
Collection locations of *Bactrocera* *trivialis* samples from Papua New Guinea and the Torres Strait Islands that were tested in this study; (**a**) Boigu Island; (**b**) Dauan Island; (**c**) Saibai Island; (**d**) Madang Province; (**e**) Central Province; and (**f**) National Capital District. Image adapted from Wikimedia Commons: Vardion, 2006, under the GNU Free Documentation License, Version 1.2.

A total of 99 specimens were assessed in this study, consisting of 21 *B.* *trivialis* and a test panel of 33 non-target species. For completeness, *B.* *trivialis* samples were included from locations throughout its native (Papua New Guinea) and invasive (Torres Strait islands) range (Fig. 3). We recognise the invasive range as regions that experience seasonal incursions of *B.* *trivialis* but are subsequently eradicated by the National Exotic Fruit Fly in Torres Straits Eradication Program^10^. To account for possible intraspecific sequence variability, samples were included from Madang Province, Central Province, National Capital District in Papua New Guinea; as well as Saibai Island, Dauan Island and Boigu Island in the Torres Strait (Fig. 3). The test panel included the same species panel included in Blacket et al.^26^; additional native Australian species genetically similar to *B.* *trivialis*, based on recent phylogenetic analysis^13^; species commonly trapped in male-lure traps in Australia; and selected species exotic to Australia that may be encountered as immatures (Table 1). In addition, we included an undescribed, morphologically similar species, *B.* sp. near *trivialis* (FFP108) that occurs in sympatry with *B.* *trivialis*. All samples used in the design and testing of these assays were morphologically identified using descriptions and keys in Drew^9^ and Plant Health Australia^31^ and subsequently confirmed through cytochrome *c* oxidase subunit I (COI) barcode sequencing (see below). Collection details and GenBank accession numbers for samples used in assay design or testing can be found in the data availability and supplementary material Table S1.

### DNA extractions, PCR and sequencing

Genomic DNA was extracted from three fly legs using the DNeasy Blood and Tissue Kit (Qiagen, Valencia, CA) as per the manufacturer’s protocol. For old, reference collection specimens, three legs were also destructively sampled, and the protocol was modified, with samples lysed in Qiagen buffer ATL and Proteinase K at 37 °C overnight. Polymerase chain reactions (PCR) were carried out to sequence LAMP target regions and confirm sample identifications. Sequencing was conducted on the Applied Biosystems ProFlex PCR thermal cycler (Thermo Fisher Scientific, USA) (see Table 3 for primer details). Products were visualised on a 1.5% agarose gel; subsequently cleaned up using the ExoSAP-IT™ Express PCR product clean-up protocol (Thermo Fisher, USA); and sent to Macrogen (Seoul, South Korea) and Australian Genome Research Facility (AGRF, Brisbane) for Sanger sequencing. New DNA sequences obtained in this study were submitted to GenBank.

**Table 3:** Loci, primers and annealing temperatures used to sequence LAMP target regions and confirm sample identifications.

Loci	Length	Primer name	Sequence (5ʹ-3ʹ)	Tm (°C)	Reference
COI barcode	307 bp	LCO1490-mod	TYTCAACAAATCATAAAGATATTGG	48.9	^33^
		Dac-COI-r	GAAAACGGRGCBGGTACAGGTTGAAC	62.0	^33^
	407 bp	Dac-COI-f	GCHTTCCCHCGAATAAATAATA	49.7	^33^
		HCO2198-mod	TGATTYTTTGGWCACCCTGAAGTTTA	55.8	^33^
EIF3L	550 bp	EIF3L-f	CCCAAGGAAAYGATCCYCAA	54.9	^18^
		EIF3L-r	GCTGACGCACTTCATCCATA	55.0	^18^

LCO1490-mod and HCO2198-mod were paired in PCR to amplify the entire 5’-COI DNA barcode region or paired with internal Dac-COI primers to amplify shorter fragments for collection samples.

### Primer design and LAMP assay reaction set-up

Reference alignments were compiled for the 5’-COI locus (~ 621 bp) and the nuclear EIF3L locus (~ 550 bp) from six species; the target: *B.* *trivialis*; non-targets: *B.* *breviaculeus*, *B.* *rufofuscula*, *B.* *parabarringtoniae* and *B.* *barringtoniae;* and *B.* *peninsularis* a species which shares genotypes with *B.* *breviaculeus* and *B.* *rufofuscula*. Alignments consisted of sequences generated in this study, together with data available on GenBank (date accessed: June 10, 2021; see supplementary material (Table S1) for GenBank accession numbers).

**Figure 4:**
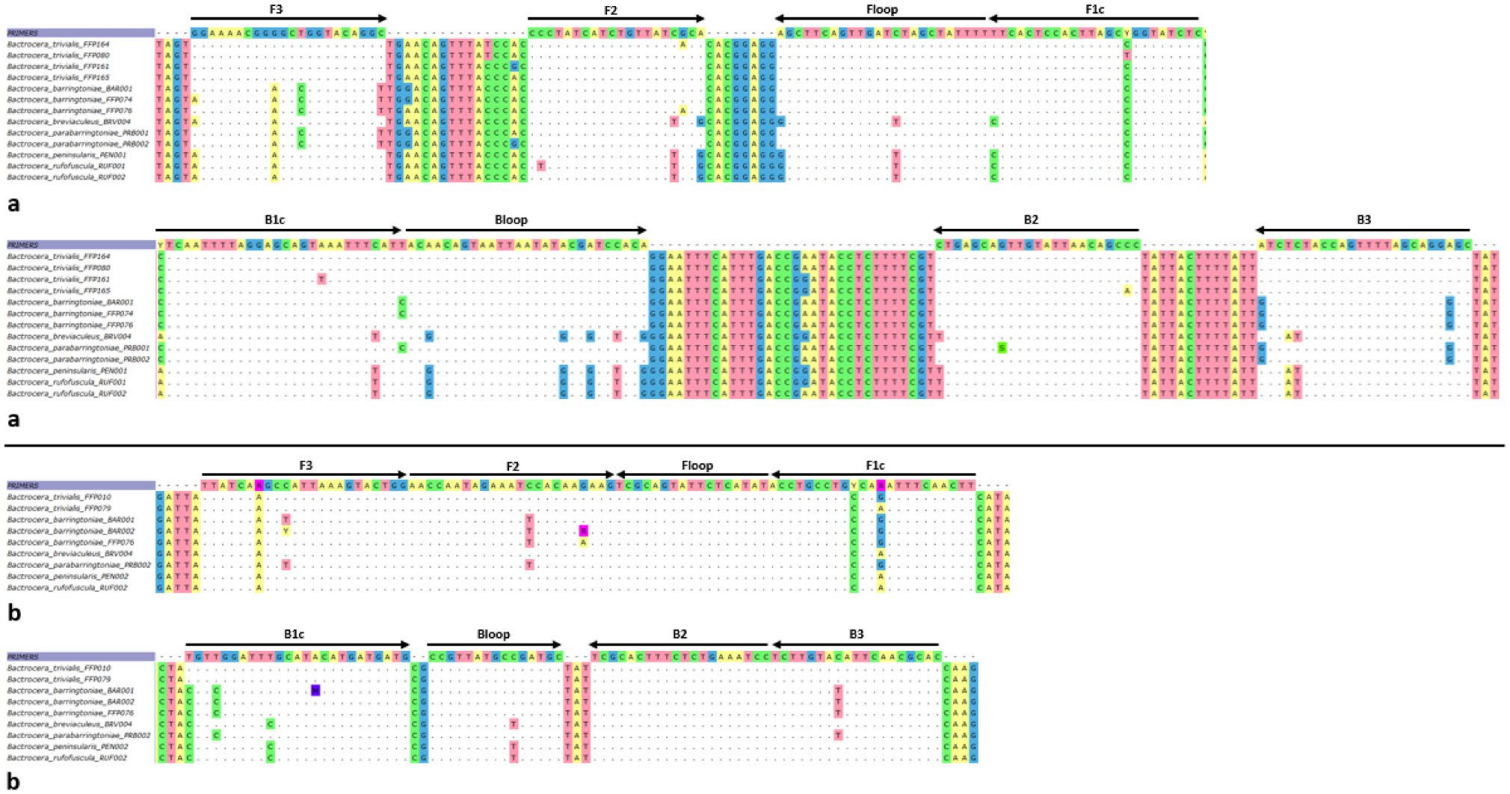
(**a**) DNA sequence alignment displaying (**a**) 260 bp region targeted in the BtrivCOI barcode LAMP assay; and (**b**) 176 bp target region in BtrivEIF3L LAMP assay. Genotypic variation in target and non-target sequences and primer orientations are shown. FIP/BIP primers target F1c + F2/B1c + B2 priming regions respectively.

Six novel LAMP primers were developed to target *B.* *trivialis* for a 260 bp fragment of COI (Fig. 4a) and a 176 bp fragment of EIF3L (Fig. 4b). Primers were designed by eye for each assay; the outer F3 and B3; inner FIP and BIP; and two loop primers: Floop and Bloop (Fig. 4a,b; Table 1). Complete sets of primers were tested for primer dimers and Tm compatibility using the ThermoFisher Multiple Primer Analyzer. Additionally, two synthetic double stranded gBlock gene fragments (IDT, USA) were designed for use as positive controls. The gBlocks were designed based on our priming regions (Fig. 4), with the addition of connecting strings of ‘g’ nucleotides between each. This design allowed us to manipulate the Tm (°C) of our gBlock fragment. We tested the BtrivCOI gBlock and BtrivEIF3L gBlock in tenfold serial dilutions to give an indication of the sensitivity of each assay^25^.

Both the BtrivCOI and BtrivEIF3L assays were run by combining 14 µL of Isothermal master mix (DR001) (OptiGene, UK) with 10 µL of primer master mix and 1 µL of template DNA. The primer master mix consisted of the three primer pairs F3/B3:FIP/BIP:Floop/Bloop that were tested at various ratios during optimisation^25^. Both reactions were run on the GENIE III (OptiGene, UK) at 65 °C for 25 min (isothermal amplification), followed by ramping from 98–73 °C at 0.05 °C/s (annealing curve analysis). Products were visualized in the blue channel on the GENIE III. We validated our methods across three independent laboratories (Queensland Department of Agriculture and Fisheries, New South Wales Department of Primary Industries and at AgriBio Victoria). We treated amplification within 20 min as positive; samples amplifying later than 20 min were reported as late amplification; and samples the GENIE III did not detect an anneal or amplification peak were considered negative.

## Supplementary Information

Supplementary Information 1.

Supplementary Information 2.

## Data Availability

Sequences generated in this study are available at GenBank. Accession numbers ON092615-ON092623 (EIF3L), and ON103574-ON103618 (COI barcode).
